# Parental Weight Perceptions: A Cause for Concern in the Prevention and Management of Childhood Obesity in the United Arab Emirates

**DOI:** 10.1371/journal.pone.0059923

**Published:** 2013-03-26

**Authors:** Abdulla Aljunaibi, Abdishakur Abdulle, Nico Nagelkerke

**Affiliations:** 1 Department of Pediatrics, Zayed Military Hospital, Abu Dhabi, United Arab Emirates; 2 Department of Internal Medicine, College of Medicine, United Arab Emirates University, Al-Ain, United Arab Emirates; 3 Department of Community Medicine; College of Medicine, United Arab Emirates University, Al-Ain, United Arab Emirates; CUNY, United States of America

## Abstract

Parental participation is a key factor in the prevention and management of childhood obesity, thus parental recognition of weight problems is essential. We estimated parental perceptions and their determinants in the Emirati population. We invited 1541 students (grade 1–12; 50% boys) and their parents, but only 1440 (6–19 years) and their parents consented. Of these, 945 Emirati nationals provided data for analysis. Anthropometric and demographic variables were measured by standard methods. CDC BMI percentile charts for age and sex were used to classify children’s weight. Parental perception of their children’s weight status (underweight, normal, and overweight/obese) was recorded. Logistic regression analyses were used to identify independent predictors of parental perceptions of children’s weight status. Of all parents, 33.8% misclassified their children’s’ weight status; underestimating (27.4%) or overestimating (6.3%). Misclassification was highest among parents of overweight/obese children (63.5%) and underweight (55.1%) children. More importantly, parental perceptions of their children being overweight or obese, among truly overweight/obese children, i.e. correct identification of an overweight/obese child as such, were associated with the true child’s BMI percentile (CDC) with an OR of 1.313 (95% CI: 1.209–1.425; p<0.001) per percentile point, but not age, parental education, household income, and child’s sex. We conclude that the majority of parents of overweight/obese children either overestimated or, more commonly, underestimated children’s weight status. Predictors of accurate parental perception, in this population, include the true children’s BMI, but not age, household income, and sex. Thus, parents having an incorrect perception of their child’s weight status may ignore otherwise appropriate health messages.

## Introduction

Childhood obesity, a global public health problem, occurs among boys and girls of all ages, socioeconomic strata, and ethnicity, with significant long term adverse health and economic consequences [1], [2], [3], [4], [5], [6]. The global prevalence of childhood obesity seems to be increasing at an alarming rate [7].

Tackling childhood obesity requires the identification of efficacious and cost-effective approaches, both in terms of prevention and treatment. Although weight reduction interventions can be highly successful, the sustainability of weight loss among children remains unclear [8], [9]. Physician awareness of childhood overweight and obesity is important, perhaps especially for children with a family history of chronic diseases, e.g., type 2 diabetes mellitus [10].

Nevertheless, sustained motivation in weight reduction requires social support mechanisms, most notably, active family support. Family environment and parental attitudes are related to childhood obesity [11], and thus seem to directly influence children’s development of (un) healthy lifestyle behaviors [12]. Thus, active parental involvement in the prevention and management of childhood obesity may well play a pivotal role in any efficacious intervention and reduction of childhood obesity [13], [14].

To involve parents, however, would require parental recognition of their child’s weight status, i.e., they should be able to recognize an ‘obese child’ as such. Several studies however have reported high levels of parental misperception in Western societies [15], [16], and incorrect perception of their child’s body weight status may also lead to incorrect assessment of the child’s eating habits and physical activity levels [17]. In a recent systematic review it was found that parental misperception is common, with as much as 62.4% of overweight/obese children being incorrectly perceived as having normal weight [18]. In some countries, parents neither understand, nor use or trust common clinical measures to identify children’s weight status [19], and less than 50% of parents accurately identified their child’s weight status [20]. Apparently, parents use alternative approaches such as visual assessments and comparisons with other children often reliant on extreme cases [19].

Population and cultural factors affect parental ability to correctly identify a child’s weight status [17], [21], [22], [23]. For example, considerable intercultural variations of what constitutes “healthy weight” have been reported [24], [25]. However, parental perceptions towards children’s weight status and its determinants in the Arab world seem to have been largely overlooked, despite the high prevalence of overweight and obesity in this population [26].

This study aims to investigate parental perceptions of their children’s weight status as compared to the actual children’s weight status based on BMI percentile charts for age and sex from the United States Centers for Disease Control and Prevention (CDC) [23], in a homogenous sample of Emirati population.

## Subjects and Methods

### Ethics Statement

The Al-Ain Medical District Human Research Ethics Committee approved all the study protocols including data collection questionnaires and consent forms. All students and their parents signed a written consent prior to their participation. For children younger than 10 years, the consent form was signed by their parents and/or caretakers. The study was conducted from January to December, 2011 in the Emirate of Abu Dhabi, United Arab Emirates (UAE).

### Subjects

Participants were children and their parents from the Abu Dhabi Childhood Obesity Study (ADCOS). This study explores the prevalence, determinants, and potential approaches to prevention of childhood obesity in the Abu Dhabi Emirate. A representative sample of students attending public schools (grade 1–12) and their parents were invited using a two-stage sampling method; with schools as primary sampling units. Out of 294 public schools, 23 were selected. 1600 students were invited, but only 1541 students and their parents consented to participate. Of these, 1440 children provided complete information. As cultural perceptions of obesity may be highly heterogeneous among nationalities, analyses were restricted to the 1035 children (out of 1440) with UAE nationality. For details of sampling methods and collected parameters, including the children’s questionnaire, we refer to an earlier publication [26].

### Anthropometric Measurements

School nurses were trained for 6 hours on the study protocols. These nurses weighed the children without shoes or heavy clothing to the nearest 0.1 Kg and measured their height to the nearest 0.1 cm on a calibrated scale with attached stadiometer (Model 769; Seca, Hamburg, Germany). A standard tape was used to measure children’s waist circumference (WC) at a point right above the iliac crest on the midaxillary line at minimal respiration and was rounded to the nearest 1.0 cm. Three separate measurements of height, weight, and WC were recorded for each student and averaged for analysis. Body mass index (BMI), was defined as the ratio of weight to height squared, i.e. weight (kg)/height (m)^ 2^. Children’s weights were classified according to BMI percentile charts for age and sex from the CDC [27], as underweight: BMI<5^th^; normal weight: BMI≥5^th^ to <85^th^; overweight: BMI≥85^th^ to <95^th^; and Obese: BMI≥95^th^ percentile. Paternal and maternal weight and height were self-reported, from which the parents’ BMIs were calculated.

### Parent Questionnaire

The parent’s questionnaire included, 1) general information about their child [gestational age, birth weight, breast feeding, number of siblings, and the position of the child among siblings of the same mother], 2) Household socioeconomic status [current marital status, education level (illiterate, primary, secondary, college/university, or graduate education), occupation, and monthly household income], 3) Family history of chronic diseases [e.g., diabetes, hypertension, kidney disease], 4) Parental perception of the child’s weight status [‘do you think that your child’s weight is underweight, normal, or overweight?’].

### Statistical Analyses

Sample size: in view of the wide range of variables and comparisons anticipated, we aimed at the largest feasible sample size within our time and financial constraints. Parental perception of their children’s weight status was compared to the BMI percentile charts for age and sex from the Centers for Disease Control and Prevention (CDC) [27]. Standard univariate and multivariate methods, such as (logistic) regression analysis were used. To understand factors affecting parental perception, notably perception of a child being overweight/obese, we carried out stepwise logistic regression of parental perception (overweight/obese *vs.* normal/underweight) as dependent variable on CDC BMI percentile, sex, child’s age, father’s education, mother’s education, paternal and maternal self-reported BMI, and household income. The same stepwise logistic regression was carried out restricted to overweight/obese children only. A p-value <0.05 was taken as the statistical significance level. Statistical analyses were performed using the Statistical Package for Social Sciences (SPSS) version 19.0 for Windows.

## Results

Ninety parents who were contacted either reported that they did not know or gave no answer to questions about the weight status of their children. Thus, the opinions of the parents of a total of 945 children were available for analysis. In most, if not all of the cases, mothers answered the parents’ questionnaires. A third (n = 312) of all children were overweight or obese, details have been reported elsewhere [26].

The characteristics of the children and their parents, by sex, are shown in **Table 1**.

**Table 1:** Descriptive analysis of the participants (children and their parents), by weight status, and sex.

Variables	Underweight/Normal		Overweight/Obese	
	Female	Male	Female	Male
	Mean (SD)	Mean (SD)	Mean (SD)	Mean (SD)
Age [years]	10.7 (3.6)	11.3 (3.6)	12.3 (3.5)	13.1 (3.3)*
BMI [kg/m^2^]	17.7 (5.3)	17.9 (5.6)	26.8 (5.5)	28.0 (6.0)
BMI percentile [CDC]	43.4 (26.9)*	38.9 (26.9)	94.3 (4.1)	95.5 (4.1)*
Gestational Age - [N = (%)]				
<37 weeks	35 (13)	34 (11.7)	22 (16.3)	19 (13.5)
≥37 weeks	234 (87)	256 (88.0)	113 (83.7)	122 (86.5)
Birth Weight - [N = (%)]				
<2.5 kg	39 (14)	50 (16.4)	14 (10.1)	26 (19.1)
≥2.5 kg	239 (86)	254 (83.6)	125 (89.9)	110 (81)
Breastfeeding - [N = (%)]				
Yes	303 (96.5)	324 (93.6)	158 (94.6)	167 (97.1)
No	11 (3.5)	22 (6.4)	9 (5.4)	5 (2.9)
Father’s Education - [N = (%)]				
Illiterate	34 (11.2)	37 (10.9)	19 (11.7)	15 (9.1)
Primary	81 (26.6)	62 (18.2)	46 (28.4)	39 (23.6)
Secondary	122 (40.1)	139 (40.9)	69 (42.6)	59 (35.8)
Tertiary	59 (19.4)	84 (24.7)	22 (13.6)	37 (22.4)
Post graduate	8 (2.6)	18 (5.3)	6 (3.7)	15 (9.1)
Mother’s Education - [N = (%)]				
Illiterate	61 (19.7)	41 (12.0)	34 (20.5)	29 (17.0)
Primary	80 (25.9)	77 (22.5)	43 (25.9)	32 (18.7)
Secondary	134 (43.4)	142 (41.5)	61 (36.7)	69 (40.4)
Tertiary	32 (10.4)	78 (22.8)	26 (15.7)	35 (20.5)
Post graduate	2 (0.6)	4 (1.2)	2 (1.2)	6 (3.5)
Household Income - [N = (%)]				
Monthly (AUD)	22,891.8 (33,375)	29,130 (38,245)	21,797 (16,995)	43,449 (108,213)

SD; standard deviation, BMI; body mass index; Significance difference between females and males are based on two-sided tests assuming equal variances with significance level 0.05. For each significant pair, the key *P<0.05 appears under the category with larger mean.

**Figure 1:**
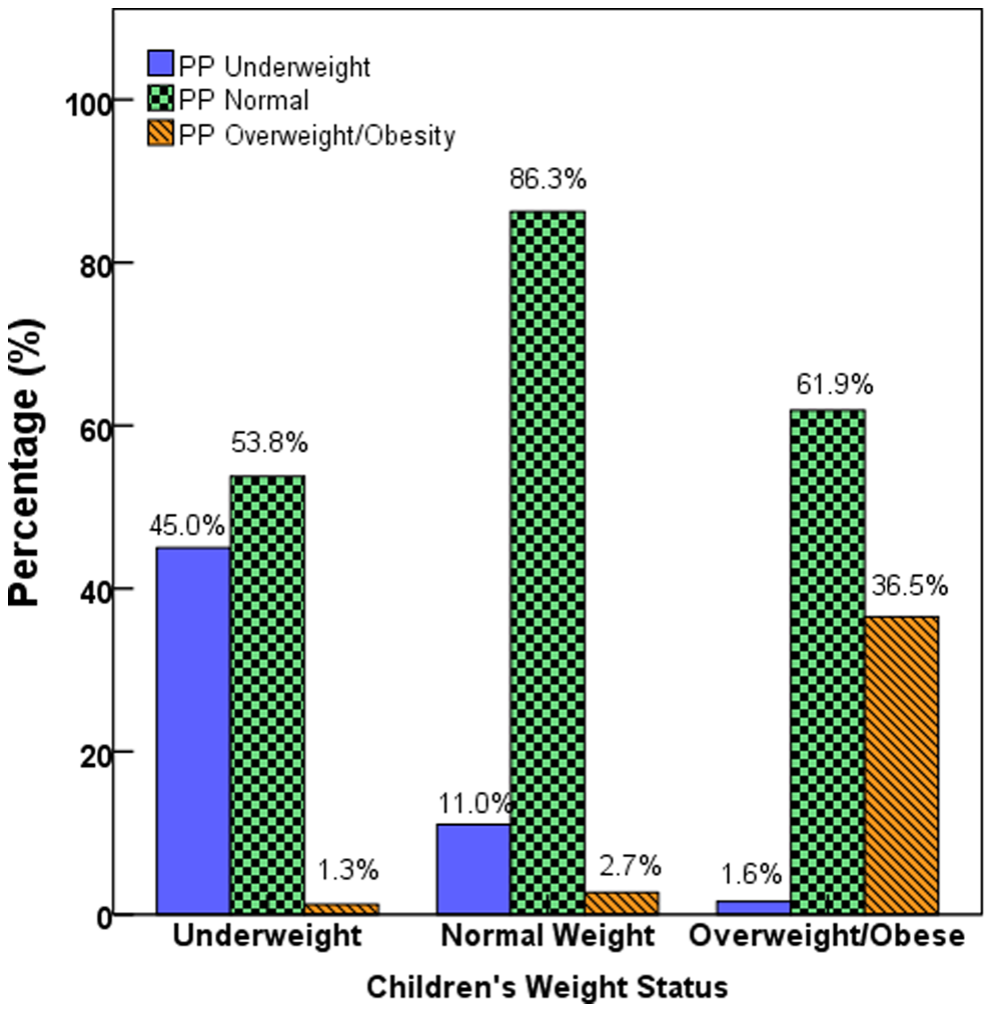
The Perceived parental perception and children’s weight status. A bar chart showing the perceived parental perception regarding their child’s weight status and actual (measured) children’s weight status categorized according to CDC percentiles for age and gender.

**Figure 1** shows bar charts of children’s true weight status by parental perceptions. Of the 945 parents 33.8% misclassified their child’s weight status either by underestimating (27.4%) overweight and obesity or overestimating (6.3%) normal weight.

A significant percentage (53.8%) of parents of underweight children classified their children as normal weight, and another 1.3% thought that their child was overweight/obese.

Many (11.0%) of parents of normal weight children classified their children as underweight, whereas only 2.7% of parents thought that their children were overweight or obese.

Not many (1.6%) parents of overweight/obese children classified their children as underweight, but most (61.9%) classified their child as of normal weight.

Three variables were found to affect parental identification of a child as ‘obese/overweight’ in a logistic regression, *viz*. the child’s CDC BMI percentile with an OR 1.073 (95% CI 1.046–1.101; p<0.001) per percentile point, and though marginally significant, the child’s age with an OR of 1.117 (95% CI 1.006–1.241; p = 0.035) per year of age and maternal education with an OR (per additional level). Of 0.711 (95% CI: 0.511–0.990; p = 0.040). Restricted to overweight/obese children, logistic regression showed that only the child’s BMI (CDC) percentile impacted parental perception with an OR of 1.313 (95% CI: 1.209–1.425; p<0.001) per percentile point.

## Discussion

In this study, we have shown a significantly high parental misperception among both parents of overweight/obese as well as underweight children. Not surprisingly, the probability that parents of overweight/obese children recognized their child’s problem increased with the degree of the child’s obesity.

We report, for the first time in the rapidly developing Arabian Gulf region, that a significant number (33.8%) of parents (overall) misclassified their children’s weight status, either by underestimating (27.4%) overweight or obese children, or by overestimating (6.4%) normal weight children. More cause for concern was our finding that the proportion of misclassification was highest (61.9%) among parents of overweight and obese children, particularly among younger children and among the borderline, incipient, overweight; a percentage in line with international findings [18]. The current results regarding parental failure to recognize the weight status of more than 50% of overweight/obese children, is of concern especially that that the prevalence of childhood obesity in this population is one of the highest in the world as previously reported [26]. Arguably, some parents may have, deliberately, opted to underestimate the weight status of their child to avoid stigmatization. Nonetheless, parents in this study were asked whether they thought that the weight status of their child was “normal”, “underweight”, or “overweight”, thus avoiding less desirable and negative weight-based terminology, e.g., fat, or extremely obese [28]. We hypothesize that cultural norms in this population may value children with rotund body shape, instead. Such cultural preferences are thought to be rooted in the extreme poverty and periodic food scarcity that prevailed in the Arabian Peninsula before the discovery of oil. Presumably, as is the case during hibernation, fat storage among humans may have had a survival value during prolonged periods of dearth.

Our results also showed that a significant percentage (54.3%) of parents of underweight children classified their child as normal weight. Underweight status is an indication of under-nutrition, and may adversely impact on the overall children’s health. Even more worrisome, is the fact that some of these children were severely underweight (data not shown), yet parents failed to recognize ‘underweight’ as a plausible health problem. Our findings indicate the importance of public education on the health risks of childhood obesity, as well as the signs and symptoms of malnutrition ‘underweight’.

In summary, a significant proportion of parents either overestimated or, more commonly, underestimated their child’s weight status. The proportion of misperception was even higher among parents of overweight/obese children. In our population, only the child’s BMI (CDC) percentile impacted parental perception. Parents having an incorrect perception of their child’s weight status may thus ignore otherwise appropriate health messages targeting obesity. Culturally appropriate interventions and awareness programs should, therefore, also specifically target incorrect parental perceptions.
